# Compensatory Strategies to Improve Functional Cognition Post-Stroke: A Review for Caregiver Empowerment and Burden Reduction

**DOI:** 10.3390/brainsci15121297

**Published:** 2025-11-30

**Authors:** Caroline Zemsky, Glen Gillen

**Affiliations:** Programs in Occupational Therapy, College of Physicians and Surgeons, Columbia University Irving Medical Center, New York, NY 10032, USA; cz2749@cumc.columbia.edu

**Keywords:** acquired brain injury, attention, caregiver burden, cognitive rehabilitation, cognitive treatment, compensatory strategies, executive function, memory, stroke

## Abstract

**Background/Objectives**: Acquired brain injuries (ABIs), such as stroke, are a major cause of disability globally and frequently affect functional cognition. Functional cognition is the ability to use cognitive processes including memory, attention, and executive functioning to perform daily tasks. When these processes are disrupted, it affects the individual in their participation, independence, and quality of life; it also places a significant burden on family members who often become primary caregivers. The aim of this review is to summarize evidence-based strategies to enhance functional cognition following strokes in an attempt to decrease the caregiver burden and improve both patients and their caregivers’ quality of life. **Methods**: This review summarizes and interprets findings using an annotated bibliography review and systematic search strategy to gather the most effective and relevant evidence-based interventions for those with ABIs and strokes experiencing memory, attention, and executive dysfunction. Studies outlining adaptive and compensatory interventions were included. **Results**: Evidence suggests compensatory strategies including environmental and external memory aids, structured routine, technological interventions, metacognitive strategies, as well as attention processing, strategy, and visual imagery training. These tactics demonstrate improvement in functional cognition domains of memory, (particularly prospective memory, i.e., remembering to perform future tasks), attention, and executive functioning after stroke and other ABIs. **Conclusions**: Effective intervention strategies can help individuals’ post-stroke become more independent in their participation and activities of daily living, leading to decreased caregiver burden and improvements in functional independence and quality of life in both patients and their caregivers. It is suggested that caregivers use these evidence-based approaches in their residential environments.

## 1. Introduction

Brain injuries represent a significant public health concern due to their high incidence and prevalence, often resulting in substantial barriers to living a healthy and meaningful life. Acquired brain injuries (ABIs), defined as injuries to the brain occurring after birth, encompass conditions such as stroke, tumors, and aneurysms. One study finds that between 64 and 74 million people are diagnosed with an acquired brain injury each year, making it one of the “leading causes of death and disability” [1]. Following acquired brain injuries (ABIs), such as strokes, individuals often experience diminished functional independence within community settings, primarily due to impairments in executive functioning, memory, and attentional control [2].

Given the high prevalence of ABIs and the resulting decline in functional independence, the impact of these injuries extends beyond the individual and place significant demands on their caregivers. Caregivers often experience burnout, distress, isolation, and “unmet needs” [3]. Additionally, caregivers are likely to have a lack of knowledge and training due to the brief time they may have to adjust to and assume their new role. Research addressing caregiver support in this context is limited, and caregivers report not knowing where or how to access appropriate resources or information [4]. Furthermore, individuals with brain injuries are often discharged home to families who have minimal resources or guidance [5]. Existing literature highlights that brain injury services tend to focus on the individual with the injury, despite the significant and interconnected impact on the family and caregivers [6].

Studies note the importance of including caregivers in the rehabilitation process and state how caregivers’ quality of life is correlated with their family member’s functional outcomes [2,7]. However, existing literature lacks the tools to assist caregivers in their role and support their well-being. After a brain injury diagnosis, family dynamics are affected and can become unhealthy [6]; thus, it is important for interventions to incorporate patient’s families and caregivers. Occupational therapists (OTs) are a primary member of the rehabilitation team and can include, or target, caregivers in their interventions. Though existing literature discusses the role of OTs in the caregiver population, most of the research addresses the caregiver burden without providing any substantial techniques to reduce that burden [4].

Due to limited family-focused interventions following an ABI, caregivers are frequently presented with difficulties in carrying out their occupational role [6]. Traditional interventions used to address caring for patients with ABIs usually take place in an inpatient rehabilitation facility by a comprehensive team of healthcare professionals [5]. Memory, (particularly prospective memory), attention, and executive functioning are three of the largely affected cognitive domains after stroke. While clinical cognition refers to the underlying mental processes assessed in standardized tests, functional cognition reflects how these cognitive abilities are applied during daily life activities. Functional cognition relates to performing everyday tasks, such as cooking, medication management, or navigating the community. Functional cognition, therefore, has a stronger correlation with participation and independence in activities of daily living (ADLs), making it a more meaningful and relevant focus for caregivers who support individuals following an ABI.

**Figure 1:**
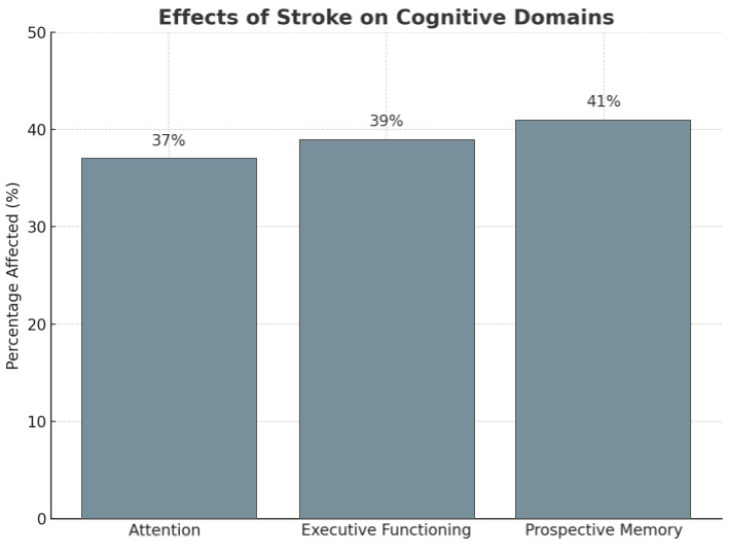
This figure illustrates the percentage of stroke survivors affected by attention, executive functioning, and prospective memory [10,11,12,13].

Figure 1 illustrates the percentage of stroke survivors affected by deficits in prospective memory, attention, and executive functioning, cognitive domains that require consistent support beyond the hospital setting. In inpatient rehabilitation, interventions targeting prospective memory include word lists, paragraph listening, visual imagery, computer assisted strategies, memory notebooks, and to-do lists. Interventions for attention include attention process training and specific skill training, and interventions targeting executive dysfunction include metacognitive strategies, problem-solving training, and management training [8]. However, these interventions are largely used by healthcare professionals and caregivers are rarely trained on or adapted by them. Existing caregiver support typically focuses on improving mental health via telehealth or group-based sessions [9]; however, these formats do not equip caregivers with resources or techniques to address their patient or loved ones’ cognitive challenges directly and may take caregivers away from their caregiving responsibilities. Effective caregiver support should be flexible, accessible, and easily integrated into daily routines. Providing caregivers with brief, digestible, written materials or guided resources, such as a caregiver manual, can better equip caregivers and increase proficiency in their role, theoretically alleviating their stress.

Recent research published in the Western journal of nursing attempted to address this problem in the context of TBIs, a subtype of an ABI. A study on “TBI transitional care intervention” called “Brain Injury Education, Training, and Therapy to Enhance Recovery (BETTER),” set out to improve patients and caregivers’ quality of life following a TBI [9,14]. With BETTER, transitional care was offered to families and patients for 16 weeks post-discharge and provided education, coping skills, and evaluation of needs to support patients and caregivers [9]. However, the intervention did not result in significant improvements in overall quality of life (QOL) [9]. BETTER was based on the United Kingdom’s TBI transitional care practices as no comparable guidelines exist for this in the United States, further revealing the gap in the literature [9]. Though BETTER is a new option for individuals and families impacted by a TBI, there are difficulties with feasibility and accessibility. Services also stop 16 weeks after TBI patients are discharged, preventing finite access to these resources. While BETTER highlights progress in caregiver-inclusive interventions for TBI, research addressing similar approaches for stroke survivors and their families is even more limited, underscoring a critical need for interventions that extend beyond the individual and provide family-centered support.

By providing an accessible overview targeting improved cognitive performance for patients, we hope to decrease caregiver burnout and unpreparedness. Having a uniform place for caregivers to go to for information may provide a viable training tool for achieving this type of support. The purpose of the present review is to analyze and interpret current research findings to aid caretakers responsible for those with brain injuries, such as stroke, in better understanding how to manage and treat their patients or family members.

## 2. Materials and Methods

The design of this review uses an annotated bibliography review and systematic search strategy to gather the most effective and relevant evidence-based interventions for those with ABIs experiencing memory, attention, and executive dysfunction.

Included studies targeted interventions on memory, attention, and/or executive functioning in populations with ABIs such as strokes. Studies that outline adaptive and compensatory interventions were prioritized, and interventions used by caretakers, healthcare workers, or anyone providing care to the brain injury population were included. Exclusion criteria consisted of strategies containing pharmacological interventions, interventions targeting cognitive deficits in non-ABI populations, outdated strategies (used over 20 years ago as these often predate current intervention models and may not reflect current, evidence-based approaches), and any studies not published in English.

Searches of PubMed, MEDLINE, and EBSCO were reviewed for interventions published in English from 2005 to present to ensure the most current evidence-based practices. Search strategy included the keywords “memory” AND “attention” AND (“executive function” or “executive dysfunction”) AND (“brain injury” or “traumatic brain injury” or “TBI” or “acquired brain injury” or “ABI” or “stroke”) AND (“compensatory strategies” or “adaptive strategies” or “functional strategies” or “compensatory interventions” or “adaptive interventions” or “functional interventions”). Covidence was used to screen titles and abstracts, and the remaining eligible studies were reviewed in depth by one reviewer (C.Z). Reference lists and studies included in systematic and scoping reviews were also reviewed when relevant. Outcome measures across the reviewed studies include pre and post interventions measures on various memory, attention, and/or executive functioning strategies. Outcome measures assessing prospective memory commonly included the Memory for Intentions Screening Test (MIST) and other neuropsychological tests as well as questionnaires including the Everyday Memory Questionnaire and the Prospective Memory Questionnaire. Outcome measures assessing attention often included the Hopkins Verbal Learning Test, the Brief Visual Memory Test Revised, and the Visual Attention Task. Executive functioning was assessed by traditional neuropsychological assessments including, but not limited to, the Stroop Color and Word Test, Controlled Oral Word Association Test, and related subtests to the Wechsler Abbreviated Scale of Intelligence-III. Measures not related to attention, memory, or any components of executive functioning (impulsivity, initiation planning, organizing, self-regulation, goal strategy training, problem-solving, etc.) were not included.

## 3. Results

### Study Selection and Analyses

**Figure 2:**
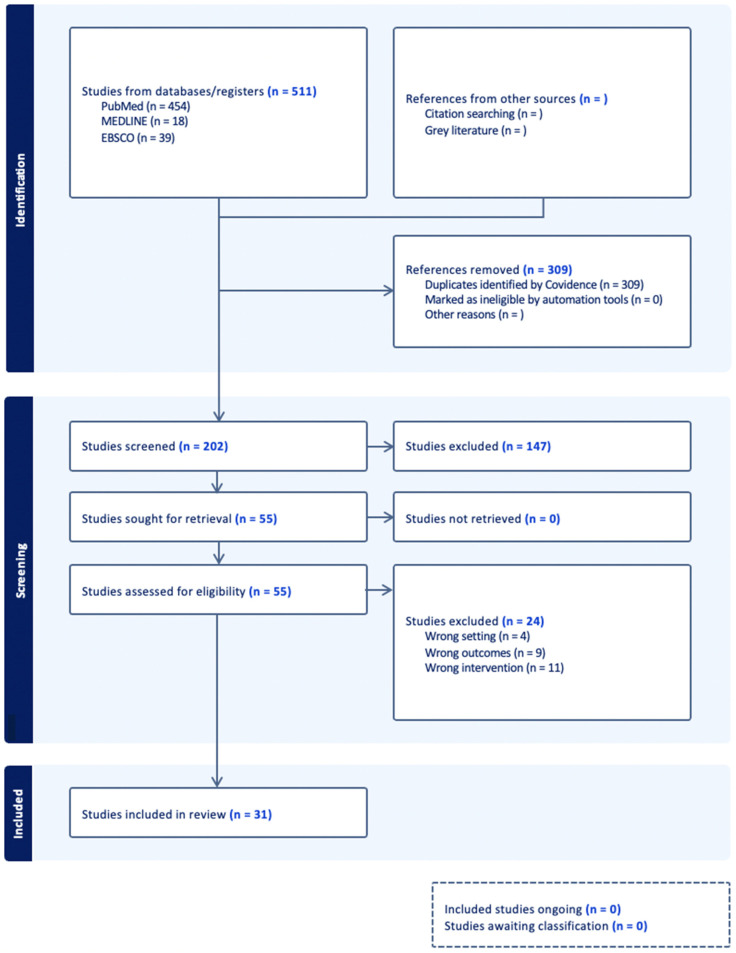
This figure illustrates the study selection process with a PRISMA flow diagram generated through Covidence.

A total of 511 papers were identified through database searches. After removing 309 duplicates, 202 studies were screened based on title and abstract. Of these, 55 full-text articles were assessed for eligibility. Following exclusions for not meeting criteria (wrong intervention, wrong outcome, and wrong setting), 31 studies were included in the final review. A PRISMA flow diagram illustrates this study selection process (Figure 2). The 31 included studies were published between 2005 and 2025, with intervention approaches targeting prospective memory, attention, and executive functioning following ABIs (Table 1).

Across studies, cognitive interventions consistently demonstrated support in one or more cognitive domains of prospective memory, attention, or executive functioning. Although the included studies addressed overlapping cognitive domains, they often measured these constructs differently. Variability in measurement approaches as well as heterogeneity in outcome measures, and intervention duration limited the ability to perform a quantitative meta-analysis. Thus, findings are synthesized qualitatively to highlight converging evidence for effecive strategies, broken down into strategies targeting PM, attention, and EF, respetively.

## 4. Discussion

### 4.1. Memory

Memory is affected in 20–50% of stroke survivors [42]. Prospective memory, which is the ability to remember to carry out tasks or intentions in the future, affects 41% of stroke survivors [10,43].

Key prospective memory strategies highlighted across several studies include compensatory tactics such as environmental cues and external memory aids. Effective environmental cues consist of structuring daily habits and routines with prospective memory tasks [36]. This can be done through implementation intentions and “if–then” or “when–then” statements, for example, “when I brush my teeth in the morning, then I will take my medications” [24,31,32,44]. Implementation intentions help individuals carry out future tasks by linking cues to an action using these types of statements [44]. This creates a mental connection between a specific situation, or cue, and a corresponding action. This then increases the likelihood that an action will occur automatically when the cue is encountered [24].

External memory aids such as diaries, notebooks, and reminder technologies including phones, Google Calendar, and pagers have all supported an improvement in memory domains, specifically in prospective memory [33,37]. Mobile phones demonstrated improvement in five study participants when they identified different domains they wished to remember such as taking medication, going for a walk, showering, applying deodorant, making lunch, brushing teeth, remembering appointments, etc. [37]. Three out of five participants were 100% successful with tasks being carried out appropriate times 100% of the time while the fourth participant improved by 92%, and the fifth improved in three reminder tasks 72%, 85%, and 83% and overall improvement from 38% pre-intervention to 96% post-intervention. [33,37]. Memory “notebooks” including diaries and Google calendar demonstrate a 15% rate improvement in prospective memory tasks when compared to no external memory aid use [33]. Specifically, utilizing Google calendar for events and reminders shows participants successful in 82% of their targeted tasks while standard diaries show a 55% success rate in remembering and achieving targeted tasks [33]. One randomized control trial supported the use of structuring tasks to environmental routines, utilizing to-do lists and daily calendar use as well as organizing and ordering tasks in improving and maintaining prospective memory [15,16].

Metacognitive approaches including various self-regulation strategies and goal setting techniques also support improvement in prospective memory functioning. For example, using sounds as cues to engage in a task or general reminders sent throughout the day support individuals in prompting task engagement [17]. Additionally, using visual imagery techniques has led to greater improvements in prospective memory overall as well as specific prospective memory outcomes like remembering appointments [17]. With visual imagery, individuals visualize themselves completing a task or visualize the cues associated with the task during task performance [17].

When combined, external memory aids and metacognitive strategies demonstrate an increase in prospective memory functioning [17]. Moreover, repetition of strategies may help in improving prospective memory performance [16]. Repetition and establishing a consistent routine, associating targeted tasks with environmental cues and implementation intentions, using external aids, implementing action directly after remembering, and using metacognitive strategies and cues as well as visual imagery all demonstrate improvements in prospective memory after they acquired brain injuries [17,25]. 

### 4.2. Attention

Attention is another cognitive domain largely affected after a brain injury. For example, after stroke, it is estimated that 20–50% of individuals experience attention deficits that affect them for years, and various attentional components are impacted including selective, sustained, divided, auditory, and visual attention processes [11,12]. Direct Attention Training (DAT) supports improvements in attention, particularly using Attention Processing Training (APT), an intervention targeting attentional deficits [26,27]. DAT is an approach targeting one’s capacity for attention through repetition and monitoring [26,27]. Through DAT, tasks are given in short intervals with simple goals. As attentional capacity improves, time increases as well as task complexity [26]. For example, caregivers can give their loved ones a piece of paper and instruct them to cross out a specific letter in a page filled with words. Research suggests that using DAT has improved attention and working memory as well as various attentional components including reading comprehension and resource allocation [11,38]. The APT–3 intervention is a virtual program that incorporates metacognitive strategies via a reflection process of participant performance. Participants review their scores and rate their perceived motivation and effort [26]. Though the APT–3 is virtual and may not be fully accessible for caregivers to implement at home, clinicians can deliver the program for individuals to use at home.

Goal setting and training also demonstrates effectiveness in improving attention. Goal Management Training (GMT) and Problem-Solving Therapy (PST) as well as mindfulness-based attention regulation training are effective strategies of goal training. In GMT, a goal is identified, information is categorized as relevant or irrelevant, and attention is focused on relevant information [28]. Mindfulness-based metacognitive strategies including a “Stop-Relax-Refocus” protocol is part of GMT and encourages individuals to implement when they become distracted or overwhelmed [28,39]. The benefit is to regain composure and focus on their initial goal [28].

When individuals utilize a “stop and think” protocol, their awareness of problem areas increase and, as a result, are better able to execute tasks [39]. Recording and identifying “absentminded slips” is part of GMT [39]. Participants’ attention improved when they were encouraged to pause upon encountering absentminded slips and difficulties and then resume the “stop-relax-refocus” protocol to refocus on their goals. Additionally, splitting up tasks into subtasks, as well as organizing and prioritizing tasks, all aided in successful GMT for increased attention [39]. Relaxation techniques, which demonstrate decreased distractions by employing mindfulness, and applying GMT to specific, individualized goals are also components of GMT that support improvements in attentional demands [39].

Compensatory cognitive training (CCT) was established as a curriculum consisting of 10 sessions in which different strategies were taught targeting several cognitive domains [18]. After completing the curriculum, improvements in attention were made on multiple neurocognitive assessments. Attention-focused strategies involved paying attention via daily practice of active listening during conversations in the home [18]. Verbal and tactile cues have also shown to be effective tools for redirecting attention and re-focusing on task engagement [34].

### 4.3. Executive Function

Executive Function comprises different components including planning, initiating, reasoning, and problem-solving, and affects 39% of stroke survivors [13,35]. Several different metacognitive strategies have proven to increase executive functioning; one such strategy is Time Pressure Management (TPM). TPM has been administered specifically to stroke patients to help combat slower processing and reaction times thereby increasing task performance [29]. Individuals are taught to better manage time pressures by making strategic and tactical decisions in order to lessen their time pressures using a 4-step: (1) analyzing, (2) preventing, and (3) managing time pressure as well as (4) monitoring their performance [29,30,40]. See Table 2 detailing the 4-step TPM strategy.

GMT and PST, metacognitive strategies improving attention, also demonstrate improvements in executive functioning [20,21]. Employing mindfulness, using the stop-relax-refocus technique, and applying training to individual goals all support an increase in executive functioning as well as attention as components of GMT [20,28,39]. A systematic review by Cicerone et al., 2019, analyzed that most studies using GMT to effectively increase executive functioning also incorporated PST and an application of GMT to everyday tasks [19,20,21,22]. The Cognitive Orientation to Occupational Performance (CO-OP) builds on metacognitive strategy training by incorporating functional components to improve executive functioning [20]. CO-OP consists of a goal-plan-do-review metacognitive strategy with the setting of a functional goal, such as organizing and managing a schedule [20]. For example, the individual will identify a goal, such a “I want to get dressed for the day and take my medication.” Then, they will plan out how to achieve this goal, breaking it down into steps such as using visual reminders in their home or setting alarms. The individual will carry out their goal using the planned techniques, and then the individual can review how the task went with their caregiver and what they can work on or improve in the future. CO-OP has shown improvements in both trained and untrained goals when targeting executive functioning, suggesting possible performance improvement in executive functioning itself [41].

Another metacognitive strategy that focuses on daily application is the Short-Term Executive Plus (STEP) program, which demonstrates effectiveness in self-reported executive functioning. The STEP program combines PST, APT, emotional regulation, and external aids to target executive functioning [22]. The STEP program utilizes SWAPS, a five-step problem solving method for individuals to carry out in daily tasks: (1) Stop! Is there a problem? (2) What is the problem? (3) Alternatives and options, (4) Pick and plan, and (5) Satisfied with the outcome? [22]. For maximal effectiveness, individuals should be taught to use the SWAPS strategy when encountering daily problems. For the emotional regulation component of the STEP program, individuals identify their triggers, reframe negative thoughts, and change any destructive views [22,23]. Additionally, APT-II was used for attention training, and planners, calendars, and to-do lists were used as external aids [22].

Overall, metacognitive strategy trainings (including self-regulation and monitoring, GMT, and PST) serve as practical strategies patients and their caregivers can implement at home to improve executive functioning [20]. By encouraging reflection and monitoring rather than direct assistance, these approaches foster autonomy while reducing caregiver burden.

## 5. Conclusions

These strategies show improvement in the domains of memory, (specifically prospective memory), attention, and executive functioning. They are practical, functional, evidence-based tools that can be applied in daily activities to not only enhance individual functioning, but ease the demands placed on caregivers. While interventions for ABIs, such as stroke, are often focused on the patient, these strategies, when implemented, can directly support caregivers. Caregivers are responsible for their patient or family members, and when daily tasks become difficult due to their ABI, cognitive impairments can further complicate these challenges. Equipping caregivers with accessible, applicable, and structured tools to decrease survivors’ challenges can also lead to a reduction in stress, burden, and lack of preparedness in their role.

When caregivers are trained on evidence-based strategies demonstrating improvements in cognitive functioning (i.e., environmental cueing, implementation intentions, external aids, metacognitive strategies, etc.) they can empower their patients and loved ones to lead more functional lives while lessening the caregiver load. In summary, strategies targeting prospective memory include the use of environmental cues, external memory aids, implementation intentions and structuring routines, repetition, and consistent schedules. These strategies play a role in increasing prospective memory functioning in individuals with ABIs such as strokes, but they can also minimize the demand on caretakers in constantly reminding, repeating, and prompting individuals while keeping track of multiple schedules and appointments. External aids like using a phone or Google Calendar for reminders, scheduling, and prompting, environmental cues and consistent routines, and implementation intentions and metacognitive strategies focused on automatic responses allow for individuals with brain injuries, such as strokes, to develop more functional independence and autonomy throughout their day; this in turn, can lead to a decrease in caregiver involvement. Attention strategies such as reflection, goal setting, problem solving, verbal and tactile cues, as well as mindfulness-based tools, including “Stop-Relax-Refocus,” develop awareness and emotional regulation that can further improve cognitive functioning and decrease emotional episodes. These strategies can help caregivers redirect attention without providing constant supervision or having to complete the task themselves. Executive function strategies including TPM, metacognitive strategies and the CO-OP, goal-oriented strategies and implementing the goal-plan-do-review tactic, as well as the SWAPS strategy can all provide relief to caregivers when implemented for patients or loved ones with ABIs such as strokes. While the individual experiencing the ABI learns to split up tasks, monitor and reflect on their performance, and apply frameworks to help with decision making, they can become more independent and less reliant on their caregivers. Additionally, emotional dysregulation can contribute to caregiver burnout, especially when they do not have or know the tools to manage emotional dysregulation. Emotional regulation strategies and the use of external aids for individuals with ABIs targeting executive functioning may then lead to a decrease in emotional outbursts, further reducing some of the burden caregiver’s face.

Of note, aerobic exercise and music therapy were two additional evidence-based strategies that were reviewed in the research. Because studies did not specify which cognitive domains these interventions targeted, instead supporting cognitive performance overall, they were not included as strategies for the purposes of this review. However, these two interventions support the growth of nervous tissue and blood vessels in the brain, which is beneficial for overall brain (and cognitive) functions [38].

These practical interventions and strategies support individuals in their cognitive recovery and can equip caregivers with the appropriate tools to foster independence. Instead of individuals with ABI relying on their caregiver to remember appointments and medications, plan and organize daily and future tasks, and manage their emotions, individuals are empowered to confront these themselves; caregivers can work alongside the individual to offer support and share responsibility rather than assuming full responsibility for all daily tasks. Empowering caretakers with these tools can lead to improved QOL not just for the individual with ABI, but also for those caring for them.

This review compiles numerous practical, evidence-based strategies to support individuals with stroke and their caregivers. However, several limitations should be noted. Most of the included interventions were validated in studies targeting patients improved performance and cognitive domains, rather than caregivers’ feelings of burden. Thus, caregiver fidelity in applying these strategies has not been evaluated. Additionally, the heterogeneity of ABIs and stroke presentation may influence the generalizability of these results and the improvement in patient performance.

Future research should focus on evaluating how caregivers implement these strategies in real-world home environments, and whether implementation translates into measurable reductions in stress, burden, and unmet needs. Developing and testing an accessible, user-friendly caregiver manual or digital tool could serve as a practical method for training and supporting caregivers in applying these strategies. Longitudinal studies following caregivers as they incorporate these interventions into daily routines would provide valuable insight into long-term feasibility, adherence, and impact on both caregiver and survivor’s quality of life.

## Figures and Tables

**Table 1:** The table categorizes the included studies based on study design.

Study Design	Number of Studies	Studies Included
Randomized Control Trials	5	[15,16,17,18,19]
Systematic or Scoping Reviews	5	[11,20,21,22,23]
Quasi-Experimental	8	[12,24,25,26,27,28,29,30]
Cross-Sectional	6	[13,31,32,33,34,35]
Case Studies and Cohort Studies	6	[36,37,38,39,40,41]

**Table 2:** TPM 4-step strategy [29,40].

Key Questions	Objectives
Are there two or more things to be done at the same time? Could I be distracted or overwhelmed?	To identify the level of time pressure associated with a given task
What can I complete prior to the task?	To avoid unnecessary time pressure
How would you respond to unexpected and overwhelming time pressure? Develop an emergency plan.	To manage time pressure efficiently and effectively
Have a backup plan ready and apply it consistently.	Encourage individuals to self-monitor while implementing TPM

## Data Availability

No new data were created or analyzed in this study. Data sharing is not applicable to this article.

## Abbreviations

The following abbreviations are used in this manuscript:

ABI	Acquired Brain Injury
TBI	Traumatic Brain Injury
OT	Occupational Therapist
BETTER	Brain Injury Education, Training, and Therapy to Enhance Recovery
QOL	Quality of life
MIST	Memory for Intentions Screening Test
WAIS-IV	Weschler Adult Intelligence Scale —4th Edition, Digit Span Subtest and Digit Symbol Subtest
DAT	Direct Attention Training
APT	Attention Process Training
GMT	Goal Management Training
PST	Problem Solving Training
CCT	Compensatory cognitive training
TPM	Time pressure management
CO-OP	Cognitive Orientation to Occupational Performance
STEP	Short-Term Executive Plus
SWAPS	(1) Stop! Is there a problem? (2) What is the problem? (3) Alternatives and options, (4) Pick and plan, and (5) Satisfied with the outcome?
